# The Oncolytic herpes simplex virus type-1 (HSV-1) vaccine strain VC2 causes intratumor infiltration of functionally active T cells and inhibition of tumor metastasis and pro-tumor genes VEGF and PDL1 expression in the 4T1/Balb/c mouse model of stage four breast cancer

**DOI:** 10.3389/fmolb.2023.1199068

**Published:** 2023-06-14

**Authors:** Rafiq Nabi, Farhana Musarrat, Jose Cesar Menk P. Lima, Ingeborg M. Langohr, Vladimir N. Chouljenko, Konstantin G. Kousoulas

**Affiliations:** ^1^ Department of Pathobiological Sciences, Louisiana State University School of Veterinary Medicine, Baton Rouge, LA, United States; ^2^ Division of Biotechnology and Molecular Medicine, School of Veterinary Medicine, Baton Rouge, LA, United States; ^3^ Global Discovery Pathology, Translational Models Research Platform, Sanofi, Cambridge, MA, United States

**Keywords:** oncolytic virus, VC2, herpes, virotherapy, breast cancer

## Abstract

**Introduction:** Oncolytic viruses (OVs) provide new modalities for cancer therapy either alone or in combination with synergistic immunotherapies and/or chemotherapeutics. Engineered Herpes Simplex Virus Type-1 (HSV-1) has shown strong promise for the treatment of various cancers in experimental animal models as well as in human patients, with some virus strains licensed to treat human melanoma and gliomas. In the present study we evaluated the efficacy of mutant HSV-1 (VC2) in a late stage, highly metastatic 4T1 murine syngeneic.

**Method:** VC2 was constructed VC2 using double red recombination technology. For *in-vivo* efficacy we utilized a late stage 4T1 syngeneic and immunocompetent BALB/cJ mouse model breast cancer model which exhibits efficient metastasis to the lung and other organs.

**Results:** VC2 replicated efficiently in 4T1 cells and in cell culture, achieving titers similar to those in African monkey kidney (Vero) cells. Intra-tumor treatment with VC2 did not appreciably reduce average primary tumor sizes but a significant reduction of lung metastasis was noted in mice treated intratumorally with VC2, but not with ultraviolet-inactivated VC2. This reduction of metastasis was associated with increased T cell infiltration comprised of CD4^+^ and CD4^+^CD8^+^ double-positive T cells. Characterization of purified tumor infiltrating T cells revealed a significant improvement in their proliferation ability compared to controls. In addition, significant T cell infiltration was observed in the metastatic nodules associated with reduction of pro-tumor PD-L1 and VEGF gene transcription.

**Conclusion:** These results show that VC2 therapy can improve anti-tumor response associated with a better control of tumor metastasis. improve T cell responses and reduce pro-tumor biomarker gene transcription. VC2 holds promise for further development as an oncolytic and immunotherapeutic approach to treat breast and other cancers.

## Introduction

Female breast cancer is the most common cancer with a nearly 40% incidence rate in the United States (Sung et al., 2021; Siegel et al., 2022). Early diagnosis and the type of breast cancer are key parameters for successful chemotherapy and immunotherapy. The clinically most aggressive type is triple-negative breast cancer (TNBC). This tumor type lacks the estrogen receptor (ER), progesterone receptor (PR), and human epidermal growth factor receptor 2 (HER2) gene amplification, rendering it resistant to conventional chemotherapy that depends on the presence of these receptors. TNBC can therefore currently not be effectively treated due to a variety of impediments including lack of targeted therapies, molecular and intratumor heterogeneity among different patients, and inability of the immune system to detect and eliminate malignant clones (Podo et al., 2010; Hurvitz and Mead, 2015; Jia et al., 2017; Al-Mahmood et al., 2018).

Several immunotherapeutic approaches have been utilized to improve survival of patients suffering from aggressive cancers (Sharma and Allison, 2015). Recently, checkpoint inhibitors have shown promise against a variety of metastatic cancers including melanoma, renal cell carcinoma, lung cancer, and bladder cancer (Vinay and Kwon, 2018). However, checkpoint inhibitors have produced only limited responses in clinical trials (Planes-Laine et al., 2019). Breast cancer has been considered poorly immunogenic rendering it less likely to respond to immunotherapies (Sisirak et al., 2012; Plitas et al., 2016; De La Cruz and Czerniecki, 2018). However, there are a number of reports indicating that certain breast cancers are immunogenic, with TNBCs exhibiting the strongest immunogenicity as evidenced by a high level of tumor infiltrating lymphocytes (TILs) (Lehmann et al., 2011; García-Teijido et al., 2016; Miller et al., 2016; Pruneri et al., 2016; Stanton et al., 2016; Liu et al., 2018) associated with increased survival (Iglesia et al., 2014). In addition, TNBCs were reported to have high tumor mutational burden and PD-L1 expression [(Thomas et al., 2018). (Wimberly et al., 2015)], suggesting that enhanced immunotherapy approaches may successfully combat these cancers. The inability to efficiently treat TNBCs as well as the emergence of drug resistant cancers raises the need for new therapeutic approaches (Scanlan et al., 2022).

Oncolytic Viruses (OVs) are widely tested as new tools to treat a variety of cancers, including breast cancers, either alone or in combination with anti-cancer drugs. Selective replication of OVs within cancers leads to tumor lysis capable of reducing tumor burden. More importantly, tumor lysis enhances anti-tumor immune responses due to the exposure of tumor neoantigens to the localized immune system, providing more efficient recognition and presentation of neoantigen signals to the innate and downstream adaptive immune responses. Also, most OVs can be engineered to express cytokines and other molecules that enhance anti-tumor immune responses (Shi et al., 2020).

The advent of molecular biology and genetic engineering has allowed the relatively easy manipulation of viral genomes to express a variety of foreign genes that enhance the immune response and allow viruses to target and replicate selectively in cancer cells. Many different viruses such as Adenovirus, Poxvirus, Coxsackievirus and Herpes Simplex viruses (HSV) have been utilized for OV therapeutic approaches (DeWeese et al., 1999; Scholl et al., 2000; Shafren et al., 2005; Garber, 2006; Gulley et al., 2008; Chang et al., 2009; Miyamoto et al., 2012; Parato et al., 2012; Andtbacka et al., 2015a; Andtbacka et al., 2015b). Currently, there are several herpes simplex virus type-1 variants that have been licensed for the treatment of cancers, while others are in phase III clinical trials. (Pol et al., 2018). Talimogene laherparevec (T VEC; also known as OncoVEX mGM-CSF and IMLYGIC) is approved by the FDA for human melanomas and is currently being investigated for a variety of other cancers (Pol et al., 2018). Recently, the HSV-1 G47Delta was conditionally licensed for the treatment of inoperable gliomas in adult and pediatric patients in Japan (Todo et al., 2022a; Todo et al., 2022b). A significant advantage of OVs is that they generally produce limited toxicity in humans since they can be engineered to selectively replicate only in cancer cells. In addition, OV mediated immunotherapy can be used to treat drug resistance tumor, providing significant advantages over other treatment approaches (Holohan et al., 2013; Pol et al., 2018). Several herpesviruses, adenoviruses, reoviruses and poxviruses as well as other viruses are therefore being pursued in preclinical or clinical studies (Pol et al., 2018).

Several HSV based OVs have been developed over the years (Menotti and Avitabile, 2020) and, in 2015, Talimogene laherparepvec (T-VEC; IMLYGIC^®^, Amgen Inc.) was approved for the treatment of melanoma (Ferrucci et al., 2021). T-VEC has the ICP34.5 and ICP47 genes deleted to attenuate its neurovirulence and improve antigen presentation, respectively. In addition, T-VEC also has an insertion of GM-CSF to promote the maturation of dendritic cells (DC) for better antigen presentation (Ferrucci et al., 2021). We developed the mutant HSV-1 (VC2) vaccine strain, which has been engineered to be unable to enter into cells and neuronal axons via fusion of the viral envelope with cellular plasma membranes. This has been accomplished by deleting two specific domains in the amino termini of glycoprotein K (gK) and the UL20 membrane protein that interact with the amino and carboxyl termini of glycoprotein B (gB), respectively. gB is the sole fusogenic glycoprotein embedded in the viral envelope and on infected cell membranes mediating virus envelope to cell membrane fusion as well as cell-to-cell fusion (Stanfield et al., 2014). VC2 replicates efficiently in fibroblasts and epithelial cells because it enters these cells via endocytosis followed by de-envelopment in the acidic endocytic compartments. However, because HSV-1 enters neuronal axons only via fusion of the viral envelope with the axonal membrane, VC2 is unable to infect neurons and establish latency in ganglionic neurons. This property renders the virus highly attenuated in mice, including SCID mice (Saied et al., 2014; Jambunathan et al., 2015; Naidu et al., 2020). We have shown in several animal trials, including mouse, guinea pig and non-human primate studies, that VC2 is a safe and immunogenic vaccine strain (Stanfield et al., 2017; Stanfield et al., 2018; Naidu et al., 2020) that confers protection of mice and guinea pigs against lethal HSV genital and ocular infection (Stanfield et al., 2014; Bernstein et al., 2019; Naidu et al., 2020).

Recently, we showed that VC2 infection of several cancer cells in cell culture resulted in significant increase of GM-CSF secretion suggesting that the observed vaccine efficacy of VC2 against lethal challenge with either HSV-1 and HSV-2 via the ocular or genital routes, respectively, may be explained by an “adjuvant” effect associated with GM-CSF production (Clark et al., Frontiers in Microbiology, In Press).

Previously, we utilized VC2 to treat melanoma in a syngeneic mouse model that showed exceptional ability to control the tumor and induced long term anti-tumor responses while generating significant immunomodulatory changes to the tumor microenvironment consistent with the generation of anti-tumor immune responses (Uche et al., 2021). The melanocytic tumors exhibited poor metastasis to the lungs and other tissues, however, limiting the assessment of anti-tumor effects of VC2 on metastasis. Herein, we evaluated the efficacy of VC2 in a late stage, highly metastatic 4T1 murine syngeneic and immunocompetent BALB/cJ mouse breast cancer model. Our data shows that OVT using VC2 induces strong systemic anti-tumor cytotoxic cell responses and improved infiltration of T cell in both primary and metastatic tumors in the lung.

## Materials and methods

### Cell line and virus

The 4T1 mouse mammary carcinoma cells were grown in RPMI 1640 medium supplemented with 10% Fetal Bovine Serum (FBS) (ThermoFisher, MA, United States) and Primocin (ThermoFisher). VC2 was constructed as described previously (Naidu et al., 2020). Briefly, the VC2 recombinant virus was constructed utilizing the two-step Double-Red Recombination protocol using the HSV-1(F) viral genome cloned as a bacterial artificial chromosome (BAC). The virus was cultivated in African green monkey kidney (Vero) cells maintained in complete Dulbecco’s Modified Eagle Medium (DMEM) (ThermoFisher) supplemented with 10% FBS.

### Model and treatment schedule

Female 8- to 10-week-old BALB/cJ mice () were purchased from Jackson Laboratories (Bar Harbor, ME, United States) and were housed in the Louisiana State University School of Veterinary Medicine (LSU-SVM) Animal Biosafety Level 2 facility. The mice were anesthetized using 4% isoflurane and oxygen was maintained at 0.5L/min. 10^5^/100 µL 4T1 cells were injected in the mammary fat pad to establish the tumor at day 0. After 10 days, when the primary tumor reached a size of 4 mm, animals were injected with 50 µL PBS/UV-VC2/VC2 in RPMI 1640 complete medium every other day for a total of 3 times. Animals were monitored every day and euthanized on day 28 by CO_2_ inhalation followed by cervical dislocation. All animal work was approved by LSU IACUC.

### Microscopic assessment of pulmonary metastasis

One Hematoxylin and Eosin (H&E) stained section including all lung lobes for each treated and untreated animal was evaluated with standard light microscopy by a blinded board-certified anatomic veterinary pathologist. The samples were subjectively classified according to the percentage of pulmonary tissue obliterated by tumor cells originating from the main mammary tumor (metastasis). Metastasis to lymph nodes and cardiac tissue within the slides were not included in this assessment. We refer to this as a semi-quantitative analysis of metastasis.

### Tissue processing and flow cytometry

Following euthanasia, the primary tumors were collected in PBS, were minced and were processed with the Mouse Tumor Dissociation Kit (Miltenyi, Germany) and gentleMACS Octo Dissociator (Miltenyi). The homogenized solution was passed through a 100 μm, 70 μm and 30 μm filter to prepare a single cell solution. This cell suspension was incubated with a pre-titrated antibody mixture for 30 min at 4°C and was then washed and fixed with 2% paraformaldehyde. The next day, samples were analyzed using the BD LSR-Fortessa™ Cell Analyzer and data was processed using the FCS Express 7 software. The following reagents and anti-mouse antibodies used for flow cytometry were purchased from Miltenyi - CD45-APC Vio770, CD11C-PE, CD24 PE-Vio770; Thermofisher - CD3-PerCP-ef710, CD4-FITC, Live/Dead Aqua dye; BD - Gr-1 PE-CF594, CD8a-BV650, gdTCR-BV711, MHCII-BV711, CD64-BV605, CD49b-BV421, CD11b BV786CD38 Percp ef710, Egr2 APC and TCRβ BV605.

### Immunohistochemistry (IHC)

Following euthanasia, tissues were immediately fixed using 10% formalin for 3 days and processed in the Histology Core Facility of LSU-SVM. For IHC, 5-μm thick paraffin sections were deparaffinized in xylene and rehydrated through graded alcohols. Antigen retrieval was performed by incubating the slides in boiling Tris-based solution (Vector Laboratories) for 1 h. Endogenous peroxidases were inactivated using 3% H_2_O_2_ for 10 min. Slides were then blocked with 2.5% Normal Horse Serum (Vector Laboratories, United States) and incubated with 1/100 diluted rabbit anti-mouse CD3 (Abcam). After overnight incubation, slides were incubated with goat anti-rabbit IgG (Vector Laboratories) followed by horse anti-goat horseradish peroxidase (HRP, Vector Laboratories). Slides were developed with Vector VIP substrate (Vector Laboratories) and scanned using the NanoZoomer Slide scanner (Hamamatsu Photonics, Japan).

### Anti-tumor cytotoxic splenocyte assay

Tumor specific cytotoxicity was measured using flow cytometric assay. Fluorescein isothiocyanate-labeled 4T1 cells were plated on a 12-well plate on the day before the experiment. On the day of the experiment, spleen was harvested and a single cell solution was obtained from each spleen. One million splenocytes were incubated with 4T1 cells for 3 days in RPMI1640 complete medium. After the incubation period, splenocytes were washed out and 4T1 cells were incubated with 1 µL 7-aminoactinomycin D (7-AAD, ThermoFisher) for 30 min at 4°C. Cells were washed, trypsinized and analyzed using the BD LSR-Fortessa™ Cell Analyzer, and data was processed using the FCS Express 7 software. Cell death was calculated as percent of 4T1 positive for 7-AAD.

### Tumor infiltrating T cell proliferation assay

Tumor infiltrating T cell proliferation assessment was done using CD3/CD28 Dynabeads. Briefly, tumor infiltrating T cells were isolated using CD4/CD8 beads (Miltenyi). After isolation, cells were labelled using the CellTrace violet dye according to the manufacturer’s instructions (ThermoFisher). Cells were activated using CD3/CD28 beads (ThermoFisher) for 3 days at 37°C in the presence of 30 IU IL-2. After the incubation, the cells were analyzed using the BD LSR-Fortessa™ Cell Analyzer and data was processed using the FCS Express 7 software. Splenic T cells from naïve animals were used as the positive control.

### RNAScope duplex

Manual RNAscope duplex was used to quantify gene transcription in 5-μm thick, formalin-fixed, paraffin-embedded lung sections. To stain RNA for specific genes, 5-μm thick tissue sections were prepared according to the ACD guideline. The slides were dried in the oven at 60°C for 1 h followed by 5 min incubation in Xylene and 1 min incubation in 100% alcohol. The slides were air dried before adding H_2_O_2_ for 10 min at room temperature (RT). All slides were then washed in distilled water and placed in boiled target retrieval agent provided with RNAScope kit. After 15 min incubation slides were submerged in distilled water and transferred in 100% alcohol for 3 min. Slides were dried in the oven and a barrier was created and proteas plus treatment was done before proceeding for RNAScope. WE used RNAScope 2.5 HD Duplex assay kit from ACDBio (Cat. no 322430) (Advanced Cell Diagnostics, United States). For all incubation with Probes and Amps, a hybridization chamber from ACDBio was used at 40°C. After protease plus treatment slides were washed with distilled water and assay probes were added followed by 2-h incubation. Following incubations Amp1-6 were added with washing in between using washing buffer provided with the kit. After Amp6 incubation red color was developed using red reagents. After red color development Amp7-10 were added with washing in between steps. Green color was developed after Amp10 using green reagents. All slides were washed and transferred in H&E solution briefly before adding 0.2% ammonium hydroxide as bluing reagents. All slides were dried before adding mounting medium. As control positive and negative control probes were used from ACDBio. The following probes were used: V-HSV186-UL48 (VP16), Mm-CD274(PD-L1)-C1 and Mm-VEGF-A-C2. All images were scanned using the NanoZoomer Slide scanner (Hamamatsu Photonics) and analyzed using Visiopharm Image Analysis Software.

### Tissue digital image analysis

To detect different cell populations within tissue sections immobilized on slides, first an app was created to distinguish the tissue from background. Empty slides were labelled as background and whole lung as tissue sample. The app was trained at ×0.5 magnification using the Decision Forest classification method with red, green and blue filters. After initial training both false positive and negative readings were optimized using a post-processing step where a minimum threshold (500,000 µm^2^) was set to define a tissue section. This app was referred to as Tissue Detection App. To detect metastasis a second app was created and trained for metastasis regions from normal tissue. From a normal and metastatic lung several parameters were defined and labelled as empty space, metastasis, normal tissue, muscle, leukocytes and RBC. This input was trained by the Decision Forest classification at ×4 magnification and several filters of Red, Green, Blue, Chromaticity red, Contrast Red Blue and H&E were used. After combined training from several control slides a post processing step was used to optimize the detection by setting a minimum threshold. To calculate metastasis from a slide, first, tissue detection was run followed by application of the metastasis app, which generated the percentage of metastasis within the tissue area. We refer to this as a quantitative analysis of metastasis as it accurately calculates the area.

### IHC and RNAScope detection

This was done similarly by generating apps or using premade apps downloaded from the VisioPharm website and running them after the tissue detection app. To calculate the RNAScope and IHC data within a metastatic area, at first metastatic area was defined by a board-certified pathologist and IHC/RNAScope apps were run only within the metastatic area.

### Statistical analysis

Statistical analysis was performed using GraphPad Prism 9 software. For analysis between three groups one-way ANOVA and Kruskal-Wallis test were performed. To compare results between two groups, the Mann-Whitney test was utilized. The statistical significance level was set at *p* = 0.05.

## Results

### VC2 replicates in cancer cells and implanted 4T1 tumors

**FIGURE 1:**
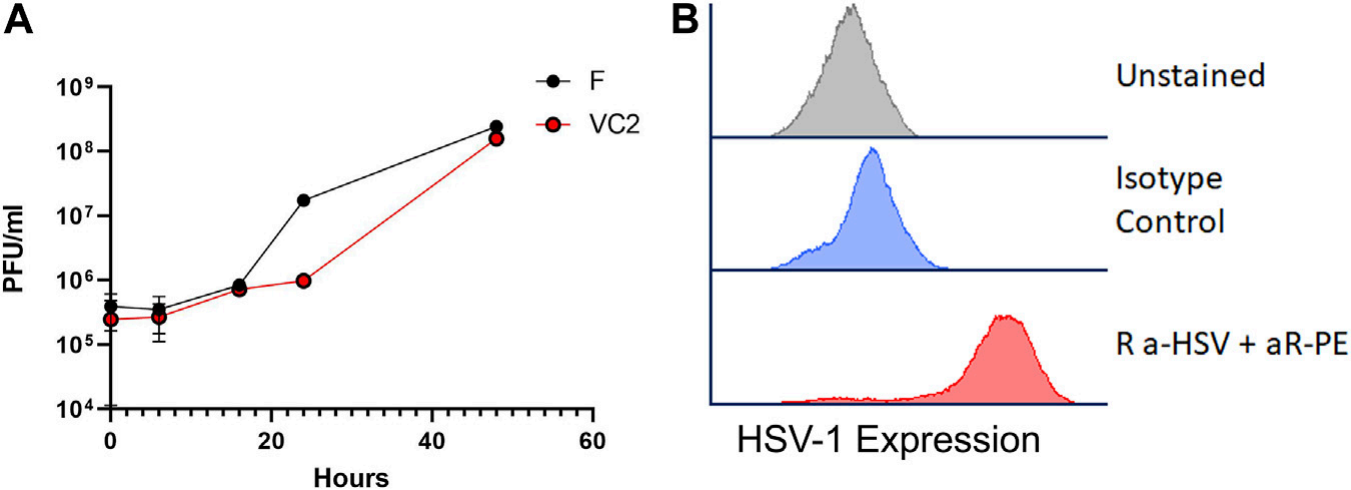
VC2 replication. **(A)** Replication kinetics of VC2 and the parental HSV-1(F) strain in 4T1 cells infected at an MOI 1. **(B)** HSV-1 protein expression detected by flow cytometry using Rabbit anti-HSV polyclonal antibody in infected 4T1 cells in cell culture.

We have reported previously that VC2 replicated efficiently in Vero and other cells, while it cannot enter neuronal axons and establish latency in mice (Saied et al., 2014; Naidu et al., 2020; Nabi et al., 2021). VC2 replicated efficiently in 4T1 cells as parental HSV-1(F) (Figure 1A) and viral antigens could be detected by flow cytometry (Figure 1B).

**FIGURE 2:**
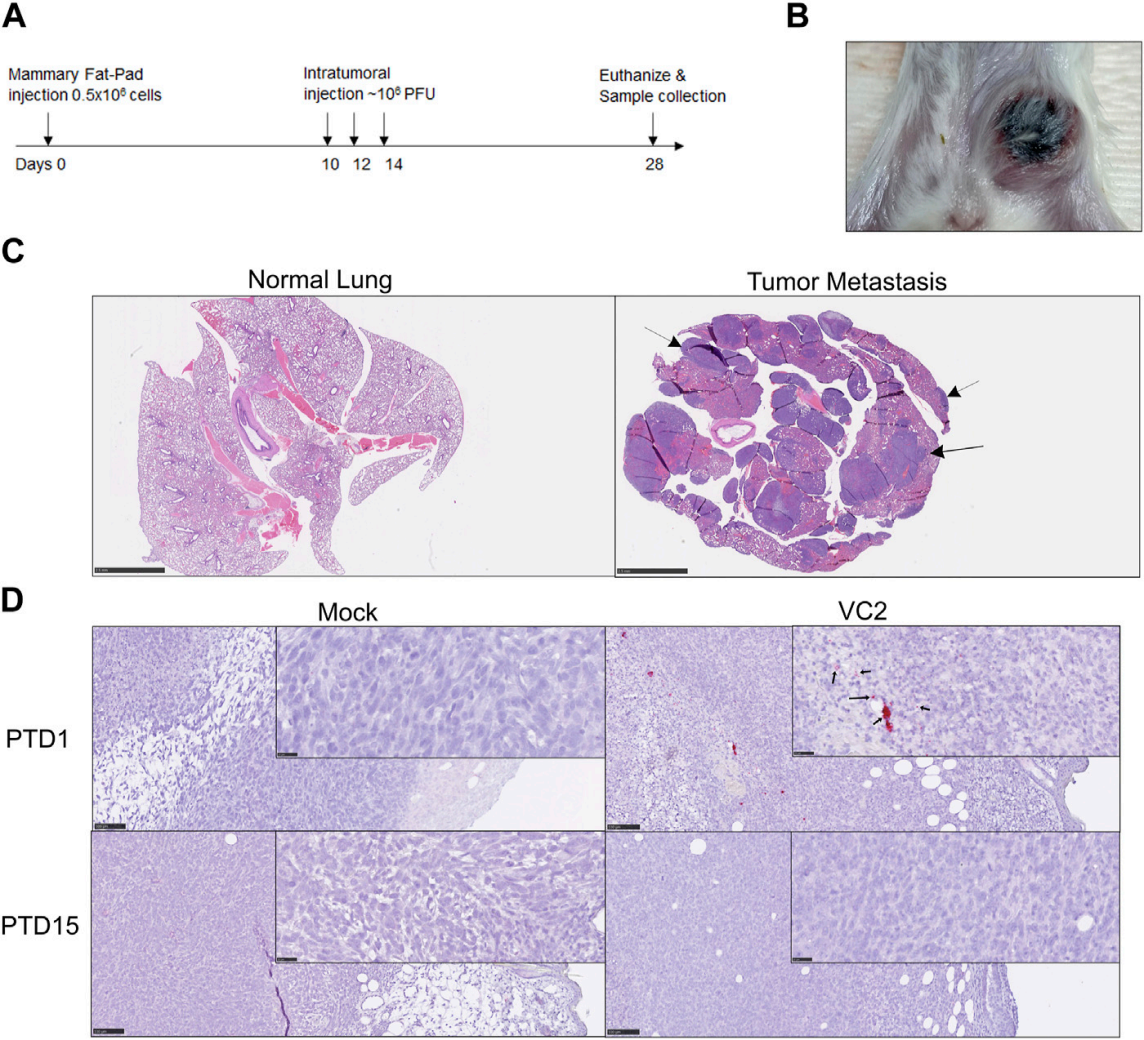
Intratumor VC2 replication and OVT treatment strategy. **(A)** Illustration of OVT treatment strategy. **(B)** Example of primary tumor at day 28 post tumor establishment. **(C)** Lung pathology; Left panel–normal mouse lung, right panel–presence of spontaneous metastasis at day 28 indicated by arrow. Magnification 0.5x **(D)** Viral replication *in-vivo*. The presence of HSV-1 VP16 mRNA transcription was detected by RNAScope (red spots indicated by black arrows) after post treatment day 1 (PTD1). Magnification 0.5x and 40X.

To investigate the ability of VC2 to replicate in 4T1-generated tumors in BALB/cJ mice, 0.5 × 10^6^ cells were inoculated in the mammary fat pad of BALB/cJ mice. The schematic of experiment is shown in Figure 2A. Tumors were palpable and could be easily visualized at 7 days post implantation. Implanted tumors grew substantially at the original site of implantation (Figure 2B) and metastasized to the lungs over the course of the experimentation. Examination of the lung at 28 days post tumor implantation revealed substantial metastasis in the form of multiple variably sized nodules (Figure 2C). Individual tumors were injected with approximately 10^5^ PFU of VC2 at days 10, 12 and 14 post tumor implantations. Using the RNAScope assay, HSV-1 VP16 mRNA was localized in multiple tissue sites corresponding to the sites of virus infection (Figure 2D).

### VC2 OVT reduces spontaneous metastasis and increases anti-4T1 cellular cytotoxicity

**FIGURE 3:**
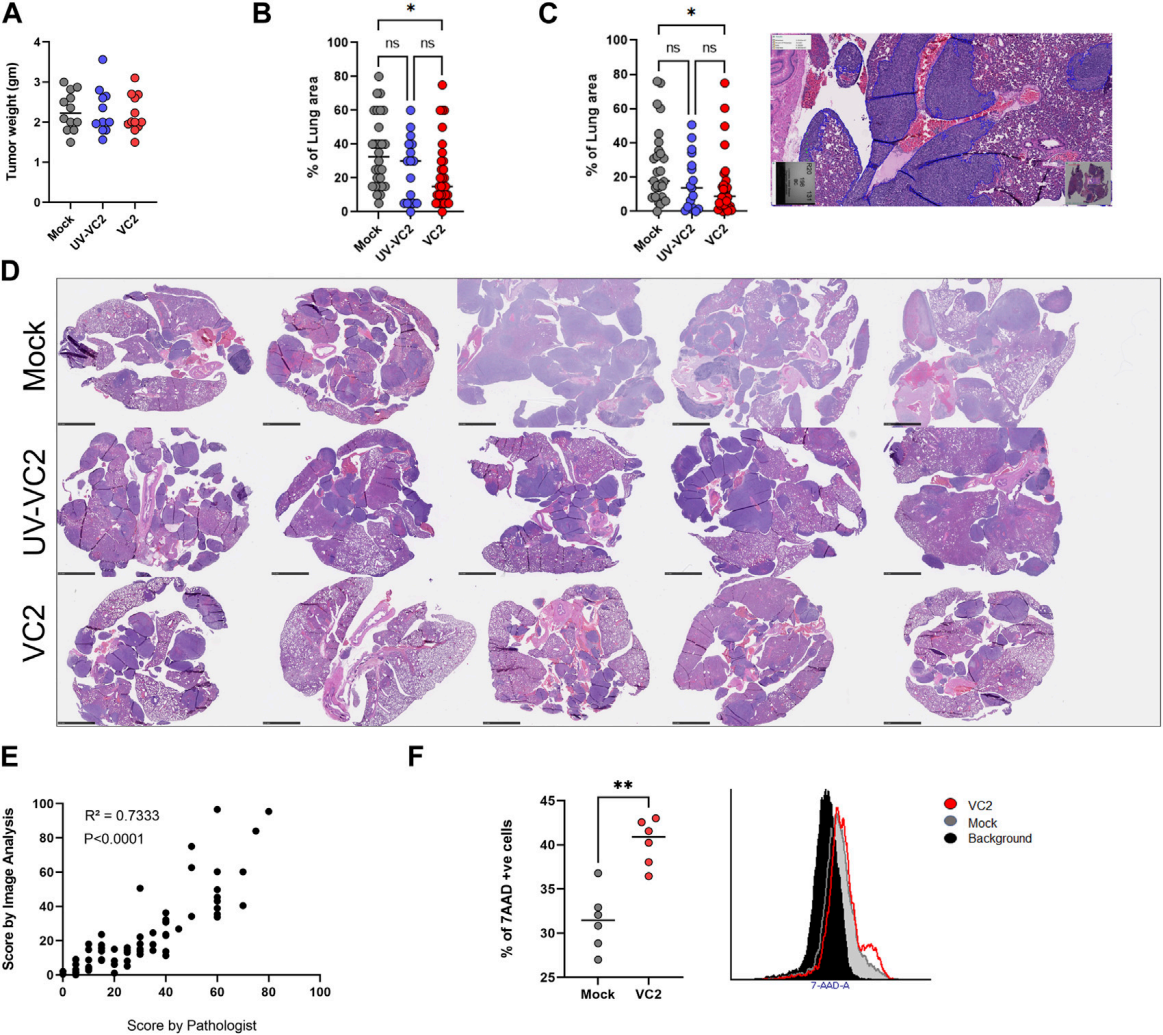
Efficacy of VC2 therapy. **(A)** Primary 4T1 tumor weights from different groups on day 28 post implantation. **(B)** Semiquantitative assessment of metastasis in the lung evaluated by pathological exam of tissue section. **(C)** Quantitative assessments of the metastatic tumor load of the lungs evaluated by image analysis. **(D)** Representative images of lung metastasis from each group of mice. Magnification 0.5x. **(E)** Correlation of metastasis quantification by a board-certified veterinary pathologist and image analysis. **(F)** Analysis of tumor-specific cytotoxic splenocytes using the 7-amino actinomycin D (7-AAD) incorporation assay. Statistical analysis between the three groups was done by One-Way ANOVA followed by post-analysis multiple comparison, N = 25 per group, pooled results from three different experiments. *p < 0.05. Statistical analysis between two groups was done by Mann-Whitney test, **p < 0.005. Correlation was calculated by Pearson correlation analysis.

The 4T1 mammary carcinoma in mice is a transplantable aggressive tumor that metastasizes spontaneously to draining lymph nodes followed by metastasis in the lung and other tissues similar to human breast cancer (Dexter et al., 1978; Aslakson and Miller, 1992). This characteristic of spontaneous metastasis can be used to evaluate the strength of anti-tumor immune responses following VC2 inoculation of primary 4T1 tumors. Examination of the VC2-treated tumors at day 28 post tumor implantation revealed that there were no significant differences in the primary tumor weights and overall appearances (Figure 3: A). Microscopic examination of the mouse lungs by a board-certified veterinary anatomic pathologist revealed that the VC2-treated animals had significantly less metastasis compared to the mock-treated control group of mice (Figure 3B). Furthermore, animals treated with UV-irradiated VC2 (UV-VC2/inactivated) had significantly higher metastasis compared to VC2-treated mice (Figures 3B, D). Quantitative analysis showed a similar significant reduction in lung metastasis in VC2-treated mice (Figure 3C) in comparison with the mock and VC2-UV inactivated mice, with a high degree of correlation with the semiquantitative data (Figure 3E).

To evaluate the systemic cell-mediated adaptive immune responses following VC2 intratumor therapy, cytotoxicity against 4T1 cells was measured using the membrane impermeant dye 7-amino actinomycin D (7-AAD) that is excluded from viable cells. VC2-treated animals had significantly higher splenocyte-mediated cytotoxicity against 4T1 cell *in-vitro* compared to the mock- and the UV-irradiated VC2-treated animals (Figure 3F).

### VC2 OVT induces T cell infiltration within 4T1 tumors

**FIGURE 4:**
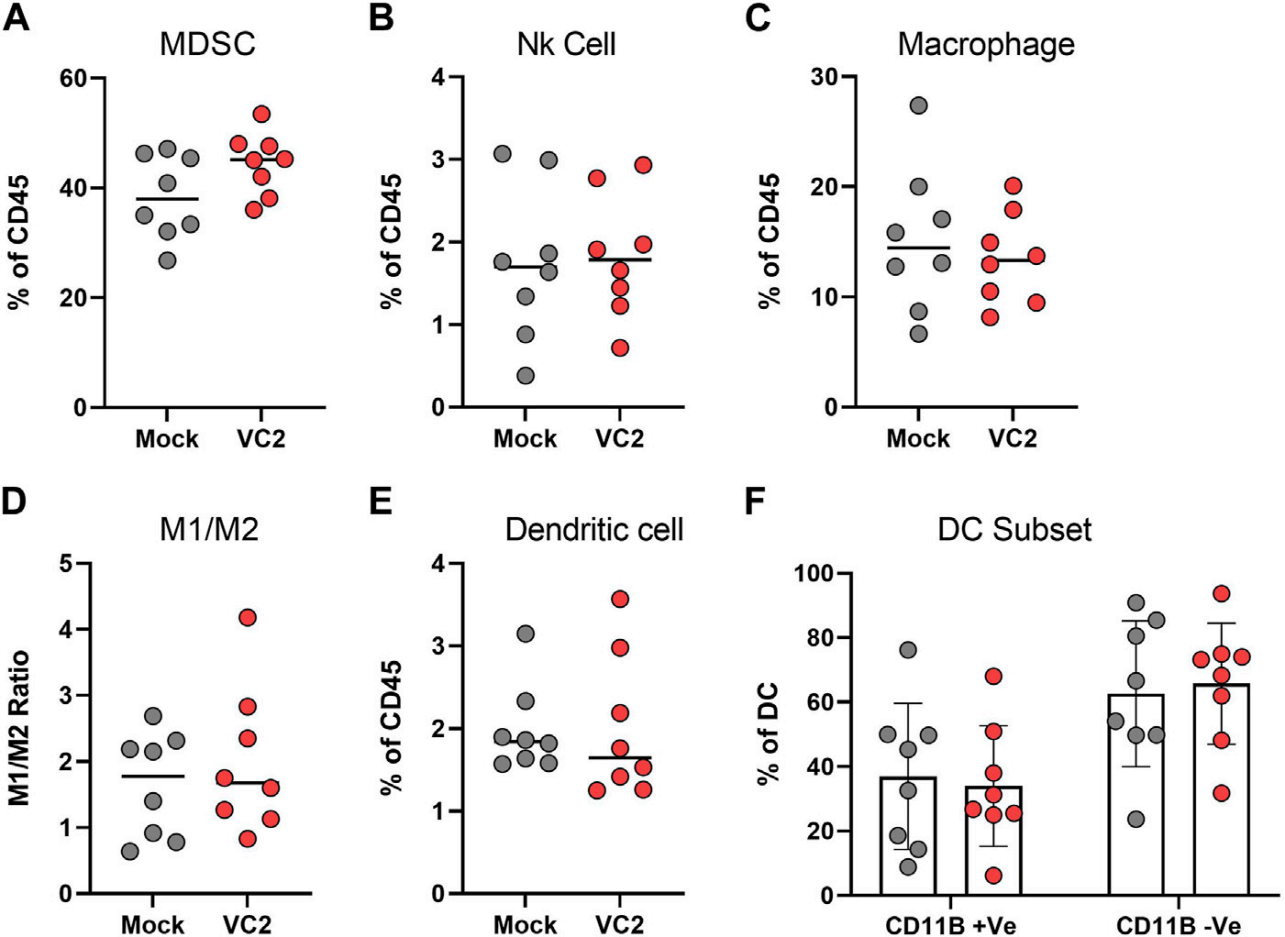
Characterization of intratumor myeloid cell infiltration. The percentage of infiltrating myeloid cells, **(A)** Myeloid derived suppressor cells (MDSC). **(B)** Natural Killer (NK) cells. **(C)** Macrophages. **(D)** M1/M2 ratio. **(E)** Dendritic cells. **(F)** CD11b^+/−^ subset of dendritic cells. Statistical analysis between two groups was done by Mann-Whitney test.

**FIGURE 5:**
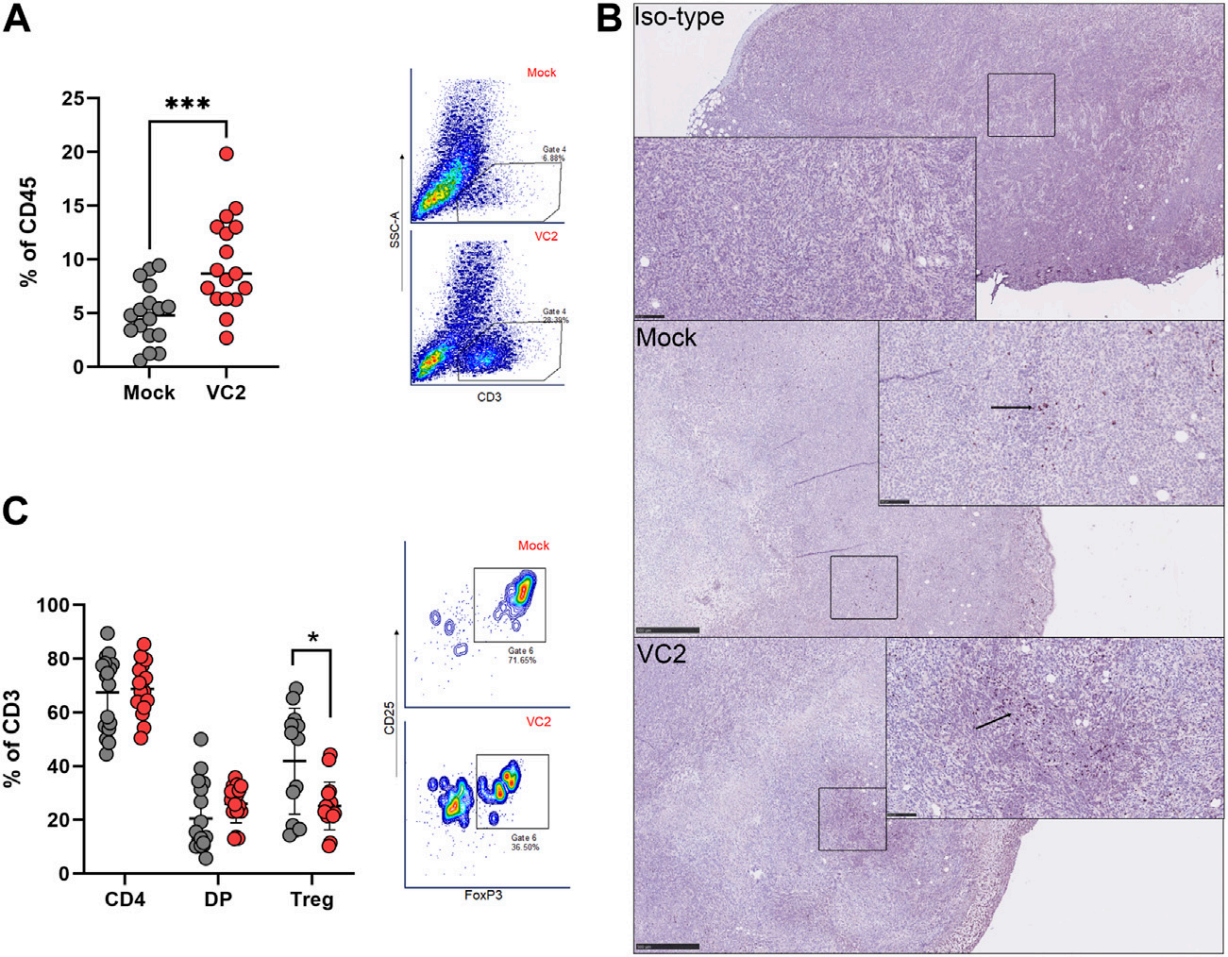
Characterization of intratumor T cell infiltration. The percentage of infiltrating T cells, **(A)** Intratumoral T cell populations in VC-2 treated and mock-treated control groups. **(B)** Representative immunohistochemistry image from each group staining with anti-CD3 antibody. Magnification 0.5x and 30x. **(C)** Intratumoral CD4^+^, CD4+/CD8+ double-positive (DP) T cells and Treg populations within the T cell compartment. Statistical analysis between two groups was done by Mann-Whitney test, *p < 0.05, **p < 0.005.

Tumor-infiltrating leukocytes were characterized to identify specific immune cell populations associated with the observed reduction in metastasis. The presence of both myeloid and lymphoid population was evaluated within the tumor on day 28. There were no differences in the myeloid compartment for myeloid derived suppressor cells (MDSCs), Natural Killer (NK) Cells, macrophages, M1/M2 macrophages, Dendritic cells and dendritic cell CD11b ± subsets (Figures 4A–F). However, a significant difference was noted in the T cell compartment, with significant T cell infiltration observed in the tumors of VC2-treated animals at 14- to 18-day post infection (Figure 5A).

T cell infiltration within tumors is beneficial in controlling tumor development and metastasis (Desfrancois et al., 2010; Kmiecik et al., 2013; Ali et al., 2014; Waugh et al., 2016; Barnes and Amir, 2017; Kurozumi et al., 2019; Xia et al., 2019; Kuroda et al., 2021) and was associated with improved outcomes (Dexter et al., 1978; Aslakson and Miller, 1992; Desfrancois et al., 2010; Ali et al., 2014; Barnes and Amir, 2017; Bernstein et al., 2019; Nabi et al., 2021; Uche et al., 2021). The IHC highlighted the presence of T cells within 4T1 tumors (Figure 5B). Further phenotyping of T cells revealed that most of the T cells were CD4^+^ and CD4^+^CD8^+^, but there was no difference between the CD4^+^ and CD4^+^CD8^+^ population within each animal group (Figure 5C). However, VC2 treated animals had significantly less T regulatory cells (Tregs) compared to mock-treated animals (Figure 5C) in agreement with previous studies from our laboratory using the mouse syngeneic and immunocompetent melanoma model (Uche et al., 2021). Several studies showed Tregs as a pro-tumor cell type because of their immunosuppressive characteristics (Spranger et al., 2013; Overacre-Delgoffe et al., 2017; Togashi et al., 2019). Thus, reduction of these population suggest an improvement in TME.

### Infiltrating T cells following VC2 treatment possesses functional characteristics

**FIGURE 6:**
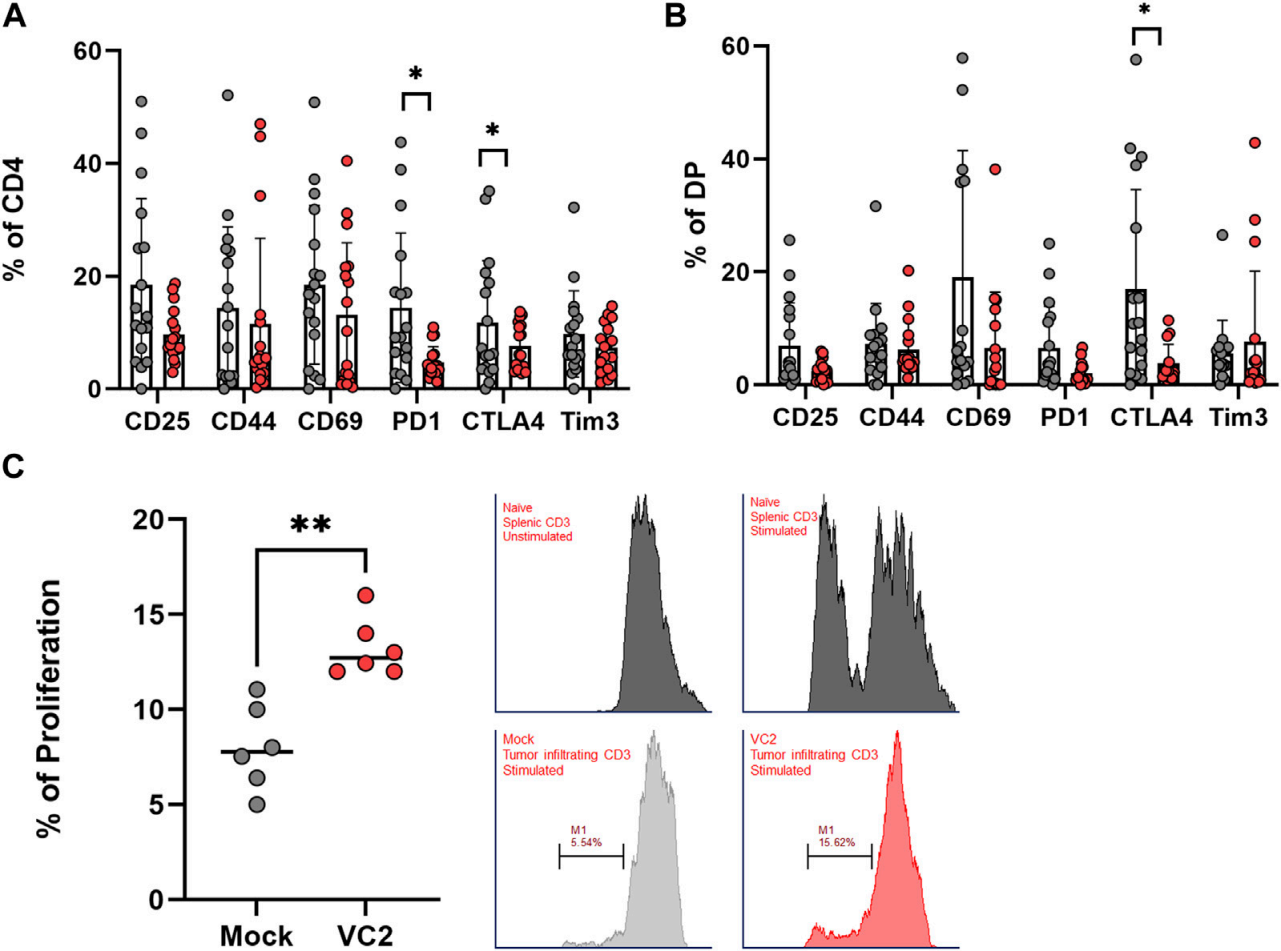
Activation and exhaustion status of infiltrating T cells. **(A)** Expression of activation and exhaustion markers on infiltrating CD4^+^ T cells. **(B)** Activation and exhaustion markers on DP T cells. **(C)** Left - Proliferation assay of purified infiltrating T cells using the CD3/CD28 activation assay. Right–Representative histogram analysis of mock and VC2 treated T cell following stimulation. Naïve T cells from spleen were used as negative control (unstimulated) and positive control (stimulated). Statistical analysis between two groups was done by Mann-Whitney test, *p < 0.05, **p < 0.005.

It is well known that the tumor microenvironment is immunosuppressive and induces T cell anergy (Webb et al., 2011; Krysko et al., 2012; Quail and Joyce, 2013; Liberti and Locasale, 2016; Palucka and Coussens, 2016). To analyze the activation and exhaustion status of infiltrating T cells, the phenotypic expression of both activation and exhaustion markers in the T cell population was investigated. Infiltrating CD4^+^ T cells in VC2-treated animals exhibited less exhaustion compared to mock-treated animals as evidenced by the lower expression of exhaustion markers PD1 and CTLA4 (Figure 6A). On the other hand, the CD4^+^CD8^+^ T cell population exhibited lower CTLA4 expression, and the population control group appeared more exhausted compared to VC2 treated animals (Figure 6B). Exhausted T cells within the tumor microenvironment generally lack the ability to respond and proliferate following T cell activation signals (Zhang et al., 2020; van der Heide et al., 2022). The ability of VC2 treatment to retain or improve T cell proliferation was tested using the fluorescent tracking dye CellTrace Violet and anti-CD3/CD8 antibodies. Following activation, the purified tumor infiltrating CD3^+^ T cells from VC2 treated animals showed significant improvement in their proliferation ability compared to control (Figure 6C). These findings suggest V2 OVT induces an improved T cell response in the tumor and retains their activation state despite the immunosuppressive tumor microenvironment.

### Improved T cell infiltration in metastatic lesions is associated with reduced pro-tumor markers transcription

**FIGURE 7:**
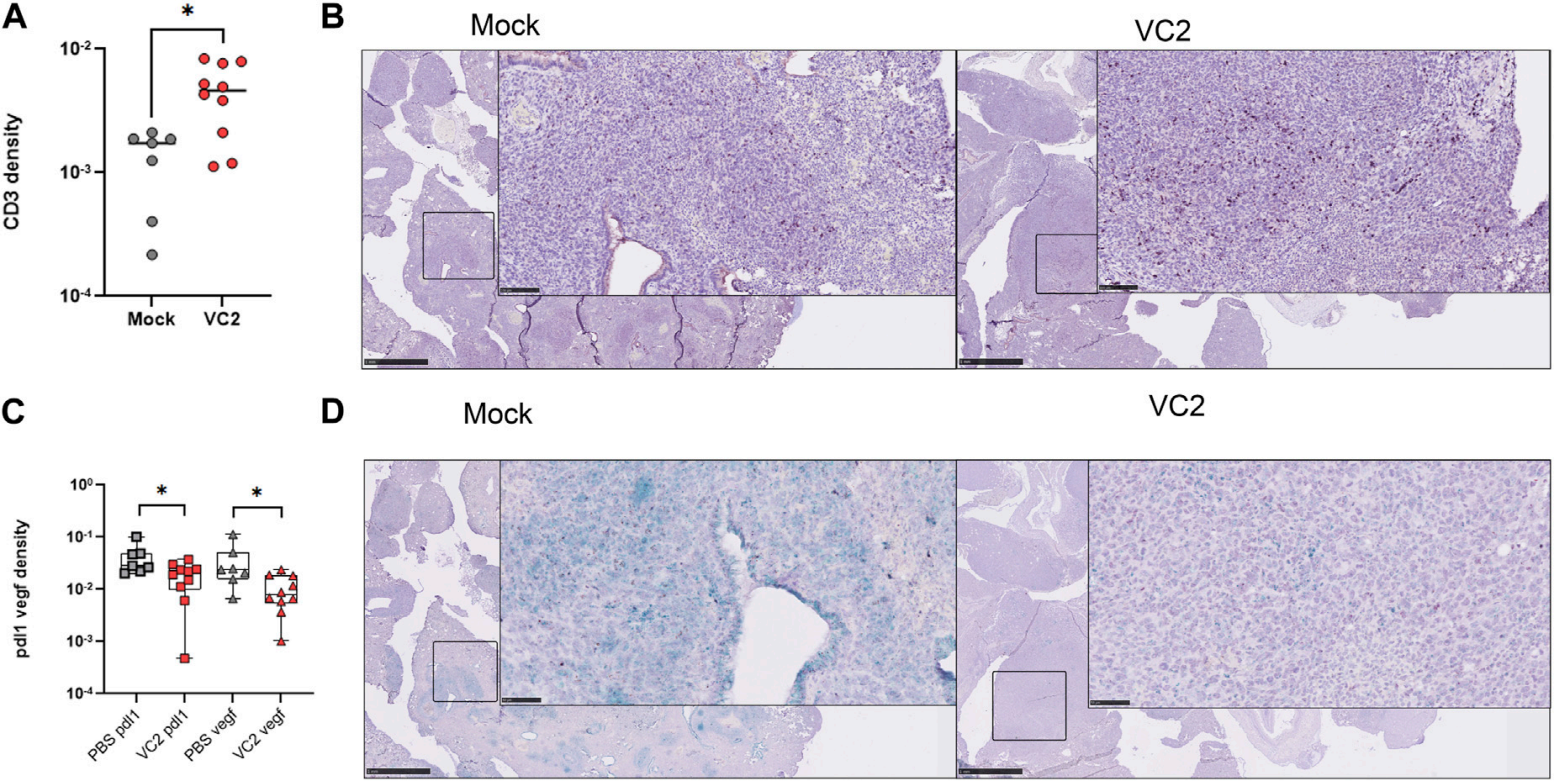
Status of lung metastatic areas following OVT treatment. **(A)** T cell infiltration in the metastatic areas in the lung determined through image analysis of immunohistochemistry on lung sections. **(B)** Representative immunohistochemistry image of the infiltrating CD3^+^ T cells in the metastatic areas of the lung. Left–mock treated and Right–VC2 treated. **(C)** Pro-tumor marker expression in the metastatic areas in the lung. PD-L1 and VEGF expressions were determined using duel RNAScope and expression was quantified using image analysis. **(D)** Representative image for PD-L1 and VEGF expression in the metastatic areas of the lung. Magnification 0.5x and 40X. Statistical analysis between two groups was done by Mann-Whitney test, *p < 0.05, **p < 0.005.

VC2 treated animals had significantly higher T cell infiltration per metastatic area of the lung (Figures 7A, B). Although T cells were identified throughout the lung, it appeared they were very concentrated in the metastatic areas especially in the VC2 treated animals (Figure 7B). The transcription of the pro-tumor markers PD-L1 and VEGF was investigated using a two-color RNAScope assay. The transcription of both PD-L1 and VEGF were significantly lower in metastatic areas of VC2-treated animals compared to mock treatment (Figure 7C). Although the transcripts of PD-L1 and VEGF were easily detectable in both mock- and VC2-treated animals, a significantly lower transcription was observed in the VC2-treated animals (Figures 7C, D).

## Discussion

Oncolytic virotherapy (OVT) using HSV-1 mutant viruses is currently utilized for the treatment of melanomas and gliomas and is actively pursued as a therapeutic approach for other cancers. Typically, these mutant viruses have been modified so that they cannot efficiently replicate in normal cells while they can replicate in cancer cells due to the lack of intact signaling pathways that inhibit virus replication in normal cells. We have developed and utilized the HSV-1(VC2) vaccine strain that is unable to enter into neurons and establish latency, while it replicates efficiently in different cell types including cancer cells. Importantly, we have previously shown that VC2 elicits strong anti-viral responses capable of protecting mice and guinea pigs against lethal challenge. Furthermore, we have shown that VC2 OVT in the mouse syngeneic and immunocompetent B16F10 melanoma model produced a significant reduction in tumor growth and anti-tumor immune responses (Uche et al., 2021). To further investigate the VC2 efficacy against highly metastatic tumors, we tested the efficacy of VC2 in the murine stage four 4T1 breast cancer model. Herein, we show that VC2 OVT of primary 4T1 tumors induces a strong systemic anti-tumor immune response resulting in significant reduction of metastasis to the lungs, associated with increased activated intratumor T cell infiltration and concomitant decrease in pro-tumor biomarkers.

VC2 replicated efficiently in 4T1 cells in cell culture, achieving similar titers at late times post infection in agreement with our previous findings. VC2 produces a small plaque size in Vero cells due to inability to spread from cell-to-cell as effectively as the wild-type parental virus (Chouljenko et al., 2009; Stanfield et al., 2014). This spread defect cannot be determined in 4T1 cells since they do not form a stable monolayer for virus plaque formation and visualization. Recently, we showed that VC2 infects and replicates fairly efficiently in a variety of cancer cells (Clark et al., Frontiers in Microbiology, In press). VC2 inability to efficiently spread necessitated multiple intratumor injections to facilitate the production of strong antiviral and anti-tumor immune responses.

VC2 OVT of primary 4T1 tumors produced a strong systemic anti-tumor immune response as evidenced by the increased anti-4T1 cytotoxicity of mouse splenocytes. In addition, a significant increase of T cell infiltration was observed within tumors, while regulatory T cells populations were reduced. Although double positive cells are not common, we found a significant portion of infiltrating T cells carry this phenotype. This contrasts with our previous observation in melanoma model. Also, these double positive cells are also present in mock infected groups suggest it is not treatment specific but rather a model specific phenotype. To make sure there is no issue with the compensation we stained these cells with different color combinations, resulting in similar observation. In addition, splenocyte from tumor bearing mice did not have these double positive phenotypes (Supplementary Figure S2) which confirmed it is tumor specific population.

We believe this increased infiltration play an important anti-tumor activity. This is in agreement with previously published studies, which reported a better prognosis associated with T cell infiltration in the tumor (Mersin et al., 2008; Marginean et al., 2010; Kmiecik et al., 2013; Ali et al., 2014; Stanton and Disis, 2016; Barnes and Amir, 2017; Kurozumi et al., 2019). Thus, improvements of T cell mediated anti-tumor control is highly desirable. Similarly, the tissue microenvironment (TME) is characterized by significant immunosuppression mediated by Tregs and myeloid suppressor T cells (Korkaya et al., 2011). The observed activated phenotype of T cells assessed by increased proliferation within 4T1 tumors and the intratumor reduction of Treg populations reveal that the VC2 OVT overcomes to some extent the immunosuppressive TME.

One of the key aspects to determine in these experiments was whether the reduction of the tumor load in the lung was associated with immunological correlates of protection in the metastatic foci. Quantifying T cell infiltration in the metastatic area within the lung is challenging as it requires both H&E to identify metastasis and IHC staining to identify T cells. We introduced image analysis steps to solve this issue and evaluate T cell infiltration. With the help of Tissue align steps we successfully aligned both IHC and H&E and calculated the T cell infiltration within pulmonary metastatic foci. Quantitative analysis indicated a significant CD3^+^ T cell infiltration per metastatic area in VC2-treated animals when compared to the mock treatment, suggesting the ability of T cells to infiltrate deep into the metastatic tumor areas. This suggests that VC2 immunotherapy generated an improved T cell population that infiltrated the tumors independent of the viral infection. In addition, the pro-tumor markers PD-L1 and VEGF were significantly reduced. These results indicate that the anti-tumor immune response was not only present within the primary tumor but remained active in metastatic foci, presumably being responsible for the reduction in the number and size of these metastatic tumors. Several immunotherapy strategies have been developed to improve the efficacy of anti-tumor T cells including CAR-T cell therapy, the use of check point inhibitors, neo-antigens vaccines, targeting metabolic pathways and oncolytic virotherapy (Esfahani et al., 2020; Watanabe et al., 2021) (Hemminki et al., 2020). VC2 OVT produces durable immune responses that prevent efficient metastasis to distant organs, showing promise for further investigations in animal and ultimately human studies.

The presence of improved T cell response following VC2 treatment may occur as a result of alternative or improved antigen presentation and processing within primary tumors. Recently, we noted that VC2 results in increased GM-CSF secretion from infected cancer cells (Clark et al., in press). Increased GM-CSF expression proximal to the injection sites may therefore be responsible for the observed T cell infiltration. It is not clear whether VC2 OVT in this mouse model induces calreticulin translocation to the cell surface, which is the hallmark of immunogenic cell death (Horton et al., 2018; Araki and Komatsu, 2020; Fucikova et al., 2020; Jiang et al., 2020; Kepp et al., 2020; Liu et al., 2020). However, it is likely that VC2 induces immunogenic cell death that alters the dynamics of neo-antigen recognition by dendritic cell and results in unique tumor specific T cell responses. As result, these T cells infiltrate the primary tumor and metastatic foci.

Future studies are needed to address the T cell anti-tumor mechanisms. Further manipulation of VC2 can be done to improve the efficacy by incorporating foreign genes that help attract and mature dendritic cells in the TME as well as others that can reduce immune suppressor cell populations. VC2 has the capacity to incorporate multiple genes toward this purpose.

## Data Availability

The raw data supporting the conclusion of this article will be made available by the authors, without undue reservation.
