# Supplementary figures and images for: The Oncolytic herpes simplex virus type-1 (HSV-1) vaccine strain VC2 causes intratumor infiltration of functionally active T cells and inhibition of tumor metastasis and pro-tumor genes VEGF and PDL1 expression in the 4T1/Balb/c mouse model of stage four breast cancer
Source: Front Mol Biosci. 2023 Jun 14;10:1199068. doi: 10.3389/fmolb.2023.1199068 (PMC10303929; doi:10.3389/fmolb.2023.1199068)

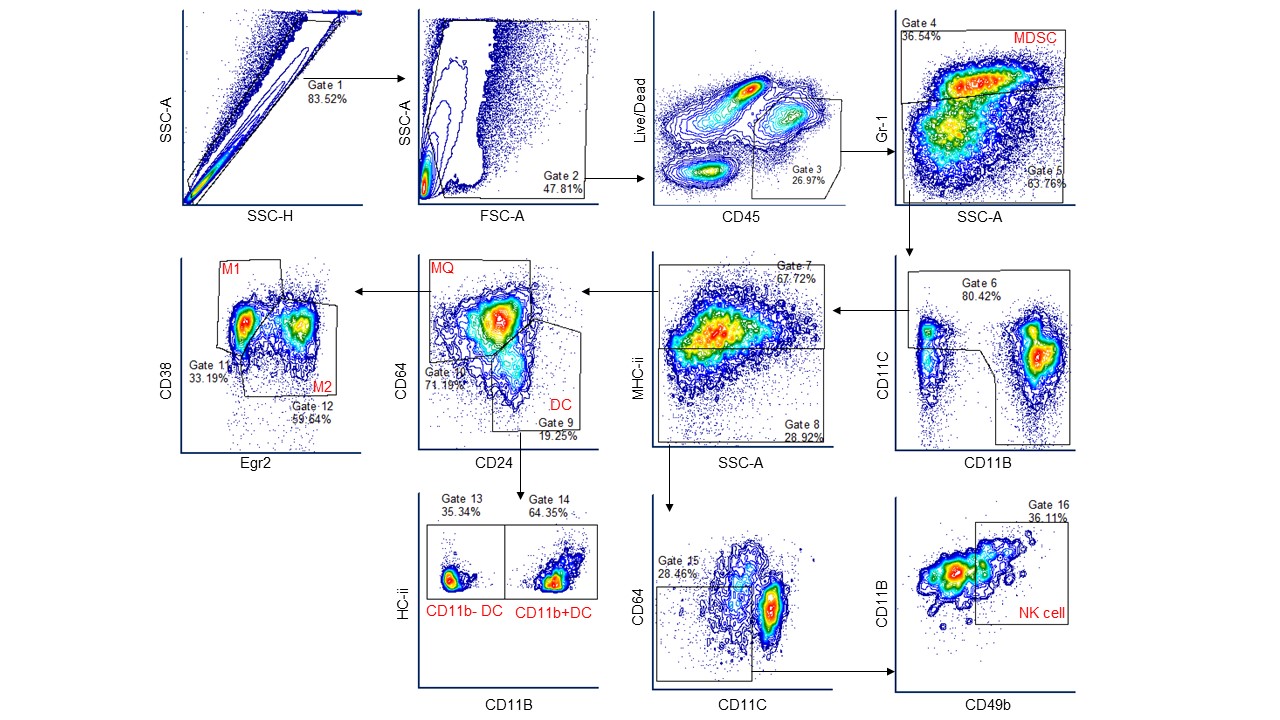

Supplement: Supplementary file 1 [file Image1.JPEG]

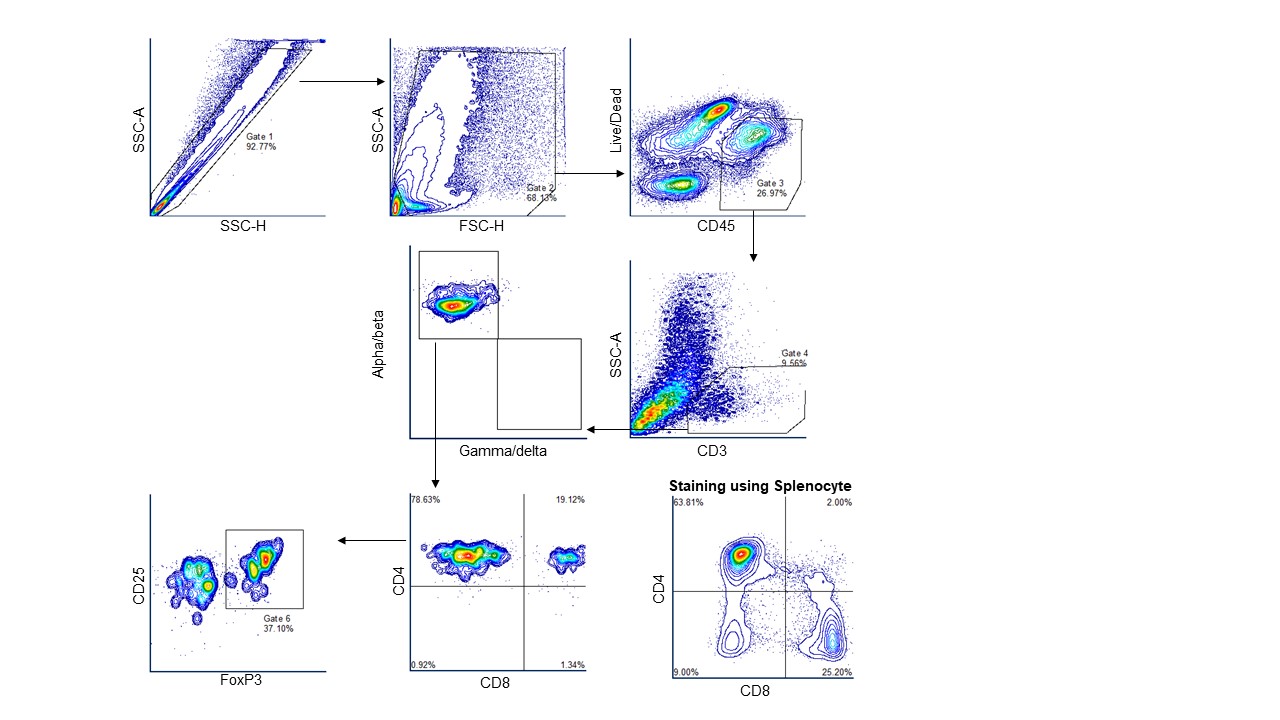

Supplement: Supplementary file 2 [file Image2.JPEG]
